# Experiences of F@ce 2.0: a person-centred intervention for home-based rehabilitation after stroke supported by digital technology — a qualitative study

**DOI:** 10.1136/bmjopen-2024-089147

**Published:** 2025-07-16

**Authors:** Kajsa Söderhielm, Malin Tistad, Charlotte Ytterberg, Susanne Guidetti

**Affiliations:** 1 Department of Neurobiology, Care Science & Society, Karolinska Institutet, Stockholm, Sweden; 2 School of Health and Welfare, Dalarna University School of Health and Welfare, Falun, Sweden; 3 Department of Neurobiology, Care Sciences and Society, Karolinska Institutet, Huddinge, Sweden; 4 Department of Neurobiology, Care Sciences and Society, Karolinska Institute, Stockholm, Sweden

**Keywords:** Cell Phone, Patient-Centered Care, eHealth, Stroke, QUALITATIVE RESEARCH, Telemedicine

## Abstract

Digital interventions may be a way of increasing access to rehabilitation after stroke, but there is limited knowledge on how they are perceived by stroke survivors. F@ce 2.0 is a home-based person-centred intervention for stroke rehabilitation that focuses on daily activities and participation in everyday life. The intervention spans over 8 weeks and uses Short-Message-Services (SMSs) to support goal achievement.

**Objectives:**

The aim of this study was to explore and describe experiences of rehabilitation with F@ce 2.0 from the perspective of people with stroke.

**Design:**

Qualitative inquiry was based on individual interviews at two time points, post-intervention and 6 months post-inclusion. Data were analysed using reflexive thematic analysis.

**Setting:**

Home-based stroke rehabilitation in Sweden.

**Participants:**

Twelve stroke survivors with mild to moderate stroke.

**Results:**

Three themes and seven sub-themes were constructed. The main themes were *‘Setting personally relevant goals requires a trusting relationship’,‘ SMSs as a tool for person-centred rehabilitation’* and ‘*Collaboration with the team is essential for resuming daily activities after stroke’*.

**Conclusion:**

Supporting goal achievement through SMS messages may increase motivation and awareness in stroke rehabilitation. The results, however, illustrate the importance of personal meetings in rehabilitation, both for setting relevant goals and for identifying goal achievement strategies.

**Trial registration number:**

ClinicalTrials.gov NCT04351178; https://clinicaltrials.gov/study/NCT04351178.

**STRENGTHS AND LIMITATIONS OF THIS STUDY**

- The study captures an in-depth view of end users’ experience of a rehabilitation intervention for stroke.
- The study contributes valuable information for future development of digital health interventions for stroke rehabilitation.
- Recruitment of participants was restricted due to administrative reasons.

## Introduction

 This paper sets out to explore experiences of F@ce 2.0, a person-centred rehabilitation intervention incorporating information-and communication technology to support goal achievement among home-dwelling stroke survivors. Stroke is one of the major non-communicable diseases (NCDs) worldwide^1^ affecting around 25 000 people in Sweden in 2022.^2^ Although a sudden incident, a stroke can be viewed as a chronic condition, leading to negative long-term consequences for functioning in daily activities and participation in daily life.^3^,^7^ In resuming daily activities, national guidelines^8 9^ highlight the importance of rehabilitation. Rehabilitation can be regarded as a process of problem solving aiming to increase participation and should be based on the perspective of the person with stroke.^10^ However, in Sweden, patient organisations have raised concerns about inequalities regarding access to rehabilitation.^11^

Digital health solutions are discussed as a way of increasing access to rehabilitation.^12 13^ In the field of stroke rehabilitation, a wide variety of digital health interventions have been tested, such as applications for computers and mobile phones, virtual-reality systems for training of motor functions as well as various devices using sensors to enable feedback during training.^14^,^18^ However, research on digital health solutions often includes specialised and sometimes expensive equipment and software not accessible to general healthcare.^15^ Rehabilitation supported by readily available information and communication technology (ICT) could be one way of orienting digital health towards accessible technology.^19^ Stroke survivors express that, although using ICT, such as mobile phones, can be challenging, it is also an important part of life and holds potential for rehabilitation.^20^,^22^ Basing rehabilitation interventions on everyday technology that is familiar to stroke survivors could thus be a feasible way of developing digital health with the goal of increasing rehabilitation access. It could further be a way of making interventions flexible and easy to adapt according to the needs of each person with stroke in accordance with person-centred practice.

Person-centred practice entails the core values of respecting personhood, building authentic relationships and basing rehabilitation on the individual’s abilities, preferences and goals while embracing all dimensions of a person.^23^ In rehabilitation, taking a stance from the perspective of the person rather than the medical condition has been described as central.^10^ To achieve person-centredness, rehabilitation teams must work in an interdisciplinary manner, setting aside professional priorities and listening to what is important to the person with stroke at that particular moment in time.^24^ Rehabilitation teams must acknowledge that participation is subjective and dependent on individual preferences^25^ not only regarding what needs to be done but also regarding what is enjoyable in life.^26^ Healthcare professionals need to truly listen to people affected by stroke and find out what their priorities are.^27^ Relationship building between stroke survivors and rehabilitation professionals is therefore essential in person-centred practice. The importance of such relationships has been underlined by stroke survivors, their family members and healthcare professionals in research exploring the experiences of rehabilitation.^28^,^30^

Person-centredness is a priority in national guidelines for rehabilitation after stroke^8 31^ and valued by healthcare professionals.^32 33^ Despite this, factors related to the person with stroke, such as cognitive and communication impairment, as well as organisational factors, make it challenging to operationalise person-centred practice in stroke rehabilitation.^27 33 34^ Employing a person-centred practice in goal-setting may be one way of shifting focus from targeting the impairment level to setting rehabilitation goals connected to life roles and what is perceived as vital or enjoyable in life.^35^ Previous research suggests that structured tools for goal setting could support person-centredness.^36 37^ Here, the Canadian Occupational Performance Measure (COPM) is often discussed.^34 38 39^ The COPM entails an interview to identify daily activities that the person finds relevant and wishes to resume or improve. Goals focused on daily activities can then form the centre of dialogue between team members and the person with a stroke to select strategies favourable for goal achievement.^40^ Incorporating ICT to further support the achievement of person-centred goals has been explored in a few earlier studies until now, with results indicating that this may be feasible.^36 41^

The F@ce 2.0-intervention was designed to enable activity-based, person-centred stroke rehabilitation using mobile phones to support goal achievement (*see detailed description under methods*).^42^ The intervention incorporates COPM as a tool for activity-based goal setting. Feasibility testing of F@ce 2.0 indicates that incorporating goals set with COPM could be one way of personalising ICT^42^,^44^ and that stroke survivors have a positive attitude regarding the intervention.^42^ There is, however, limited knowledge on how stroke survivors experience ICT-supported interventions. Furthermore, they are not a homogenous group, and the ability to handle everyday technologies is often related to stroke severity.^45 46^ The aim of this study was therefore to explore and describe experiences of rehabilitation with F@ce 2.0 from the perspective of stroke survivors who have participated in the intervention.

## Methodology and method

This research was nested within a larger study aiming to evaluate effects of the F@ce 2.0 intervention (*manuscript submitted*). A study protocol has previously been published.^47^ To explore stroke survivors’ experiences of the intervention, a qualitative methodology was used where the first author interviewed a subgroup of participants. The point of departure was that participants’ views of rehabilitation with F@ce 2.0 were formed in interaction between experiences of the rehabilitation, previous knowledge and experiences, and the dialogue with the researcher. Furthermore, the researchers bring with them extensive experience of clinical work and research within the field as well as in-depth knowledge about the F@ce 2.0 intervention. Based on this, Reflexive thematic analysis^48^ was chosen as the method of analysis.

### The F@ce 2.0-intervention

F@ce 2.0 is designed to support stroke rehabilitation focusing on daily activities. In the intervention, daily activities are understood as everything that the person wants or needs to do in everyday life. The intervention, which spans over 8 weeks, is person-centred and supported by Short-Message-Service (SMS). The intervention is further based on two overall therapeutic strategies for person-centred practice: using the patient’s lived experience as a base for rehabilitation and enabling new experiences through performance of valued daily activities.^47^ The intervention is structured in four stages: (1) F@ce-to-face meeting, (2) Assessment, (3) Collaboration and (4) Evaluation.^49^ The initial *face-to-face meeting (stage 1*) between the person with a stroke and the therapist forms the basis of the relationship building essential for person-centred rehabilitation. In a dialogue between the patient, therapist and, when relevant, also a family member, the patient is encouraged to describe earlier abilities, roles and habits. *Assessment (stage 2*) aims to identify important daily activities that the person with stroke wants, needs or is expected to perform but is not able to do due to the stroke. To facilitate a shared view, the participants are encouraged to describe their activity performance. Performance of an activity can also be filmed and watched together. A discussion around goals is initiated using the COPM.^40^ *Collaboration (stage 3*) is achieved through goal setting and through a dialogue on possible strategies for goal achievement. Collaboration is further developed through daily SMSs to support working towards the goals between in-person meetings. This contact consists of a morning SMS with a reminder of the set activity goals and a follow-up SMS in the afternoon asking the person with a stroke to rate performance of the activities set as goals (from 1 to 5, where 1 means ‘not so good’, and 5 ‘carried out the activity very well’). If SMSs indicate that goals need to be reformulated or revised, goals can be renegotiated during the intervention period. *Evaluation (stage 4*) of the goals is carried out after the 8-week programme, and during this stage, a plan is made for continued rehabilitation.

### Study setting

This study was nested in a larger research programme designed to evaluate the F@ce 2.0-intervention in a pragmatic cluster-controlled trial.^47^ The research programme was situated in Sweden where healthcare is publicly funded and organised by 21 regions, yet provided by both public and private operators.^50^ National recommendations state that rehabilitation should start during the acute phase and that it can be continued in different settings, both within specialised rehabilitation clinics or home-based teams and within the primary care system.^8^ The recommendations also state that rehabilitation should be multidisciplinary. In the research programme, 12 home- and neurorehabilitation teams from three Swedish regions participated, of which seven were intervention teams delivering the F@ce 2.0-intervention as an add-on to usual rehabilitation and five were control teams delivering rehabilitation as usual, that is, multidisciplinary rehabilitation based on the need of the person with stroke. All rehabilitation teams consisted of at least one occupational therapist and one physiotherapist, whereas access to other professions such as nurses, speech and language therapists and social workers differed between teams. Before delivering the intervention, all intervention teams participated in a series of four training workshops (8 hours in total) to learn how to deliver the intervention. Due to the COVID-19 pandemic, the workshops were delivered online. During these workshops, the teams had the opportunity to pose questions to members of the research team.

### Participants

Inclusion criteria for F@ce 2.0 were a stroke diagnosis, enrolment in one of the participating units, the ability to participate in 8 weeks of intervention, the ability to formulate and express activity goals in Swedish or with the assistance of an interpreter and the self-reported ability to use a mobile phone. No further exclusion criteria were applied. Rehabilitation teams were asked to inform any new patient who fulfilled the inclusion criteria about the intervention. If willing to consider participation, a researcher from the group contacted the stroke survivor to give further information and obtain consent. Data collection within the larger research programme lasted from February 2021 to April 2023.

For the present study, a purposive sampling among participants from the intervention group was planned to gain a broad representation of contexts.^51^ Data collection started in December 2021. All participants eligible for the post-intervention interview after this time point and up to the finalisation of data collection within the research programme were invited to participate (n=14). One declined due to fatigue and one due to the loss of a near relative, leaving 12 participants in total, 5 women and 7 men, from all three regions. Participants were between 48 and 92 years old and a majority (n=9) were living together with a partner. A majority (n=11) had had a mild stroke according to the Barthel Index^52^ (see table 1).

**Table 1:** Participants and goals

Gender	Age range	Stroke severity	Rehabilitation goals
Male*	>80	Mild	Improving fitness through taking daily walks
			Being able to walk to the local centre and back
Female	>80	Mild	Being able to see friends
			Being able to walk 7000 steps daily
Male*†	67–80	Mild	Being able to stand up for longer periods without feeling fatigued
			Being able to take walks two times daily
Male	45–66	Mild	Achieving activity balance through resting 1 hour at lunchtime
			Waking up feeling rested
			Improving fitness by walking 10 min every day after work
Female	>80	Mild	Writing without making errors
			Being able to talk well when eager
			Being able to read as fast as before
Male*	>80	Moderate	Getting out of bed without help
			Being able to go to the toilet without help
			Using assistive devices when dressing/undressing
Female	67–80	Mild	Being able to use the left hand to open drawers and cupboards
			Having a more functional gait pattern
			Being able to put the left arm on the table without compensating
Male‡	45–66	Moderate	Walking safely with support
			Improving hand-/arm function by doing daily exercises
			Moving around without the wheelchair indoors
Male	>80	Mild	Perform the exercise programme to increase balance and strength
			Walk up and down stairs daily
			Take walks outdoors
Female*	67–80	Mild	Dressing the lower body and using the left hand in the activity
			Being able to eat with the fork in the left hand
			Being able to walk indoors without an assistive device
Female	67–80	Mild	Being able to walk to the laundry room without an assistive device
			Being able to carry a bag of clothes to the laundry room
			Being able to open the door to the laundry room
			Standing up while cooking
Male	67–80	Mild	Being able to walk to the post box
			Putting on pants without help
			Being able to do grocery shopping without help

To protect anonymity age is presented as range. The lower range (45–67) is based on the age of retirement in Sweden.

* A family member participated in the interview.

† Participated in post-intervention interview only.

‡ Participated in follow-up interview only.

### Research team

The research team consisted of three experienced researchers (SG, CY and MT) and one doctoral student (KS) with extensive clinical experience of stroke rehabilitation. SG, CY and MT, all active researchers within the field, have published research based on both qualitative and quantitative data. KS is a speech language therapist, SG an occupational therapist and CY and MT are both physiotherapists. All researchers had in-depth knowledge of the F@ce 2.0 intervention.

### Data collection

Data were collected through semistructured interviews. Each participant was interviewed on two occasions: after completing the F@ce 2.0 intervention and again at follow-up 4 to 6 months later. Due to circumstances unrelated to their stroke, one participant was able to partake in the post-intervention interview only and one participant only took part for the follow-up interview. The follow-up interview was conducted to explore whether and how participants had continued to resume daily activities after the intervention period. Interviews were conducted by KS and took place in participants’ homes. For four participants, a significant other was present during the interview and took an active part in the interview by clarifying questions and reminding the participant of details (see table 1, participants). Interviews followed an interview guide with open-ended questions focusing on the rehabilitation process with F@ce 2.0 such as “can you tell me about the first meeting with the team” and “can you tell me about your challenges in daily life” (onlinesupplemental files 1  2). Before the second interview, KS transcribed the initial interview verbatim and did a thorough reading to ensure that aspects relevant for that specific person were followed up. All interviews started with an open question about life after stroke to encourage participants to freely describe their situation. During the interviews, KS aimed to let participants speak uninterruptedly and add their own reflections while at the same time keeping the focus on experiences of rehabilitation with F@ce 2.0.

### Data analysis

Data analysis followed the six steps of Reflexive Thematic Analysis.^48^ During data familiarisation (*step 1*), KS transcribed all interviews verbatim and read through the transcriptions. KS also performed initial coding and code refinement (*step 2*) using the computer software Atlas.ti. Initial coding was open but continued analysis was focused on quotes linked to the aim of the study, that is, experiences of rehabilitation with F@ce2.0. Initially, codes from the first and second interviews were analysed separately. Due to a substantial overlap, codes from both occasions were, however, used jointly. During the process of code and theme refinement, KS, MT and CY had an ongoing dialogue and met regularly. Preliminary themes were created from codes perceived as related in terms of the issue discussed, such as difficulties handling a mobile phone or appreciation of the rehabilitation team (*step 3*). From these initial themes, the final themes were created around matters that stood out as pertinent in relation to rehabilitation with F@ce 2.0 (*step 4*). After the development of initial themes, KS and SG read through all the interviews and themes were further refined (*step 5*) and named (*step 6*) through discussions within the whole research group.

### Ethical considerations

This research followed the Helsinki declaration, and ethical permit was obtained from the Swedish Ethical Review Authority (dnr. 2012/101-31/1, 2013/1845–32, 2020–01124). A member of the research group not involved in data collection gave participants verbal and written information about the research project and obtained consent. Considering the risk of communication being affected by symptoms related to the stroke, care was taken to ensure that participants understood the consequences of participating and were aware that they could opt out at any time.

### Patient and public involvement statement

Data from a previous feasibility study were used as part of modelling the intervention. The researchers also met the team members several times to listen to experiences from their everyday work. This informed the content of the interview guide.

## Results

**Figure 1:**
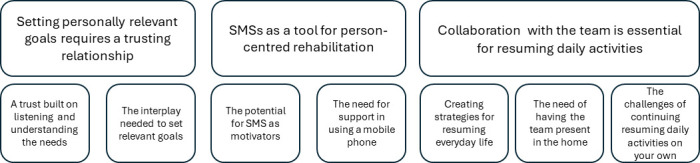
Themes. SMSs, short-message-services

Three main themes and five subthemes were constructed during analysis of the interviews. The main themes were as follows: Setting personally relevant goals requires a trusting relationship, SMSs as a tool for person-centred rehabilitation and collaboration with the team is essential for resuming daily activities (see figure 1).

### Setting personally relevant goals requires a trusting relationship

Forming a trusting relationship with the rehabilitation team or individual team members was described as positive and was essential for setting goals that were personally relevant as well as for coming up with strategies for goal achievement. Two subthemes formed the basis for this overarching theme: a trust built on listening and understanding the needs and the interplay needed to set relevant goals.

### A trust built on listening and understanding the needs

Being listened to was depicted as intrinsically positive and was strongly connected to relationship building. Participants expressed that trust was built when team members tried to understand them, conveying a genuine wish to offer support in this new life situation. One participant even described being heard and seen by his occupational therapist as a new and affirmative life experience *"she has like seen how I work (.)that’s what’s so good (.)… I have never met a person like that in my whole life” (male, 45–66)*. Trust in the relationship was also enhanced by mundane factors relating to communication and continuity, such as knowing how to reach the team or having a plan for team visits. Here, participants for whom communication with the team felt natural and flexible depicted how this contributed to a sense of satisfaction, as one participant stated: “*And if there is something I can just call them and talk to them*” (*female, 67–80*). Later in the recovery process, having a way of contacting the team again if needed and being aware of future rehabilitation possibilities seemed to contribute to a sense of security.

Participants’ reflections also illustrated how the relationship was strengthened by the feeling of being in capable hands. They perceived the team members as knowledgeable, professional and able to answer their questions and guide them in handling life after stroke. Some participants depicted a strong connection to a specific team member, describing how that person was able to explain what rehabilitation after stroke meant or say the right things to motivate them in trying something new. One participant, for instance, talked about how he felt that he had learnt a lot from his physiotherapist *“she is wonderful and she teaches and is quite a serious young person” (male, >80*). Another participant expressed how he felt when the physiotherapist gave him the right kind of feedback: “*she has managed to motivate me and helped me….(.) Positive feedback and positive vibrations that have made me feel that training has been fun” (male, 45–66)*.

### The interplay needed to set relevant goals

The interplay between participants’ feelings of being listened to and their experiences of being in competent hands seemed to be a key to identifying goals that were personally relevant. Participants depicted how the team sought to pinpoint what activities felt most important to resume but also expressed feeling unsure about what was achievable and needed the teams’ expertise.

The recollection of having set goals together with the team differed between participants. Most remembered partaking in a dialogue on matters that felt essential at that point in time, but in the follow-up interviews, there were examples of the teams not fully having understood the priorities of the stroke survivor. In connection to this, participants talked about the healthcare professionals as experts who had both the theoretical knowledge and the professional experience needed to provide them with suggestions of goals and/or activities. They described knowing what they wanted to be able to do and trusting the team to help them in getting there. As one participant stated: ”Y*es I have managed to get them (my wishes) through so to speak but I have such poor knowledge about the stroke… (.) ….I just have the wish that they should (.) that we should do things that are good for the stroke*” (male, 67–80). At the point in time when the second interview was held, most participants no longer had any contact with their rehabilitation team. Looking back on their rehabilitation, a few participants talked about situations where the interplay between their needs and the competence of the team had been halting. They felt that the team had not fully understood their needs or wishes and talked about aspects missing in rehabilitation. One participant, for instance, described how voice changes related to the stroke affected her regular meetings with friends at the boule club. She felt that the team had been too quick to dismiss this issue. “*I thought it was a bit curt (.) because I could talk but I don't have the same voice” (female, 67–80)*. Reflections like this mainly came up during the second interview, when participants had a longer experience of life after stroke.

### SMSs as a tool for person-centred rehabilitation

Participants described how the SMSs could be a tool for them in rehabilitation, both for being reminded of the goals and strategies and for increasing motivation. Managing the SMSs was, however, described as challenging by some of the participants. The overarching theme regarding SMSs as a tool was based on two subthemes: the potential for SMSs as motivators and the need for support in using a mobile phone.

### The potential for SMSs as motivators

Participants described how the SMSs reminded them of their rehabilitation goals. This was clear from the second interview where many participants still referred to activities related to the goals, although the intervention had ended 4 months ago. Participants also talked about being pushed in a positive way to pursue their goals. Reflecting on goal-fulfilment before answering the afternoon SMS was depicted by some participants as fun and motivating. They talked about wishing to complete their rehabilitation assignments and how reporting back to the team led to a sense of achievement. The strive to ‘fill the quota’ could further aid in keeping up with less motivating strategies for achieving the goals, such as performing a daily exercise programme: “*This training inside on the floor* (.) *I could take it or leave it … As I said I did it anyway (.) I had to fill the quota”, (male, >80*). Another participant talked about receiving few home visits and felt that the SMSs worked as a form of contact with the team: *“well this is a form of contact that comes at least, that they show me that they are out there somewhere”, (female, 67–80)*. This sense of presence was described by another participant as motivational: “Well *you get that reminder every morning (.) and it follows you all day. I have to do this and that in order to fulfil it”, (male, >80*). When the SMSs stopped coming, some participants found it harder to keep up with activities: *“But if I hadn't had the text messages, I wouldn't have done it to the same extent as I did. Now I have sort of fallen back a bit.”, (male, 45–66)*. The SMSs were also described as invoking a sense of the team being present, as one participant stated. “*Well, it’s a form of contact that comes anyway, that they show that they are there somewhere*.”, (*female* 67–80).

Although potentially motivating, participants also described ways in which the system with the SMSs could be improved. One thing discussed was that reporting back by rating performance of goal activities on a scale from one to five was lacking in nuance. Some participants expressed that they wanted to explain their ratings, especially when experiencing difficulties in performing the agreed activities and having to rate lower than the day before.

Another matter that came up during the interviews was the degree to which the SMSs became a two-way communication with the team. Some participants described that the team had seen the ratings and used them as a basis for dialogue. One participant, for instance, talked about having a more negative view of her progress compared with the team and how this became apparent through her daily ratings. She depicted how this had led to discussions around different aspects of progress in rehabilitation. “*I didn’t want to set it too high at once so that it could go totally the wrong way later (.) backwards (.). Then the girls from the team said that aren’t you rating a bit too low, but I said that I don’t think that I am*.” (*female, 67–80*).

For participants where the team did not comment on the daily ratings, the text messages were seen as unrelated to the rest of their collaboration with the team and sometimes as a burden. Participants’ comments also made it clear that goals could lose their relevance during the intervention period. When goals were not updated or changed, constantly being reminded of them was described as repetitious and lacking variation. *“Yes I felt it was the same questions, nothing else in particular that they asked but it was the same questions every day.”, (female 67–80)*.

### The need for support in using a mobile phone

Although owning a mobile phone was a criterion for participation in the project, not all participants knew how to send and read SMSs. While some participants described how they, after initial difficulties, were able to master the messages on their own, others reported that their need for support was overlooked by the rehabilitation team. Participants depicted receiving help only when the team noticed that the daily ratings were not sent according to the set plan. “*I got one of those messages later that I hadn’t been in contact or that I hadn’t done what I was supposed to …. But then one of these girls came here and knew how I should do it”*, (*female 67–80)*.

Once the team had recognised and met the need for support in handling the mobile phone, most participants could start using SMSs. However, for a few participants, handling a mobile phone was a major obstacle. Instead, a preferred way to support goal achievement could be a checklist on the wall. A few participants also highlighted that receiving messages from an unknown number felt unsafe and suggested that the team could have helped them to enter the number used in the intervention into their telephone book.

Having difficulties handling the mobile phone was, however, not necessarily a barrier. For two participants, the difficulties led to a natural collaboration with a family member who helped them in receiving and sending the SMSs. This, in turn, led to a joint reflection on activity performance and became a way of creating awareness of progress. Family members also felt that reminders were taken more seriously when coming from the team members through the SMSs and not from them.

### Collaboration with the team is essential for resuming daily activities after stroke

Participants described how life after stroke involved struggling to handle everyday life and understanding what they needed to do to

‘get better’ and resume daily activities. Collaboration with the team was described as essential in this effort and entailed support in doing activities and developing strategies. The theme regarding collaboration was based on the following three subthemes: Creating strategies for resuming everyday life, the need of having the team present in the home and the challenges of continuing to resume daily activities on your own.

### Creating strategies for resuming everyday life

A strong incentive for engaging in rehabilitation was getting back to the old self and being independent in daily activities. The loss of life roles, for instance of acting as the primary manager of the household, was described as sad and frustrating. In trying to recapture them, it could be hard to know where to start. Here, participants described how the team helped them identify activities that could be performed independently if carried out in a new way. In relation to this, participants discussed the motivation for using strategies and the varying ways in which strategies were developed in collaboration with the team and others. Strategies were sometimes depicted as a tool in recapturing valued life roles altered by the stroke. One woman, for instance, recalled how the team advised her to use the kitchen counter as a workspace when emptying the dishwasher and how dividing this task into two steps meant that she was able to perform it independently. Through collaboration and dialogue, the team could help identify possible strategies based on previous habits. One participant described how she had always used a calendar during her working life and how this was now a way of compensating for her impaired memory. She also recounted that the team had suggested combining this strategy with visual clues to ensure successful activity performance: *Yes but they thought it was good to write things down and so on (.) … And then I can also do it like if I want to do the laundry the next day I can get everything out and put out detergent and have everything ready (.) then I know what I’m supposed to do.”, (female>80*).

Participants also talked about coming up with strategies on their own, although discussions with the team could sometimes act as a spark in this process. Other sources of inspiration for inventing strategies were family members or assistant nurses from the home care service. One participant said that she found the strategy for putting on a bra suggested by the team much too complicated but described that she had continued working on solving this problem on her own and in the end had come up with her own way of performing the task. Feelings regarding the need for collaboration with the team in finding strategies were mixed. While some participants depicted being left alone to solve their problems, others expressed that they had always been problem solvers and said that it felt more natural to try things on their own before asking for help.

### The need for having the team present in the home

Receiving hands-on support in the struggle to resume daily activities was portrayed as essential by participants. It was clear that the collaboration around strategies and the SMS reminders of rehabilitation goals was not enough. This wish of having the rehabilitation team present seemed connected to their role as experts but also as compassionate motivators. Participants expressed that doing daily activities in a new way or following exercise programmes on your own could lead to feelings of uncertainty due to the lack of feedback. “I *would have like to have a bit more (.) maybe that someone says that this would be a good thing to do*.” (*female, 67–80*).

For some participants, the presence of the team also meant doing activities that they did not perceive as safe or even possible to do on their own. One participant who lived on the third floor with no elevator talked about being strongly motivated to recapture the ability to walk up and down the stairs and to go outside again, but not daring to do this without the team present. “I had no idea whether it was at all possible to walk the stairs.*” (male>80*).

Being active was also affected by pain and fatigue limiting the energy to work towards rehabilitation goals. In relation to this, the collaboration with the team through one-on-one meetings was described as central for keeping up motivation. Participants described the team as necessary for getting things done and described how the presence of the team made them put in extra effort. Motivation could come not only from encouraging comments but also from the team explaining why and how different activities could lead to goal achievement. One participant described that having the team present was the only way of getting exercise programmes done. *“Eh when you have them here you get things done (.) to do the training and develop it and (.) You always want to impress the teacher” (male 67–80)*.

### The challenges of continuing to resume daily activities on your own

The importance of collaboration with the team was sometimes more apparent after the rehabilitation period had ended. During the second interview, some participants conveyed a feeling of insecurity about the next step, related to an unfulfilled need for advice. They expressed a sense of not doing everything that they should, both in terms of being active in daily activities and in terms of doing different kinds of training. Here, a few participants felt that there should have been a stronger focus on daily activities during the rehabilitation period with the team. One perspective depicted in relation to this was that having the ability to do something and actually doing it could be two different things, and that regaining function did not automatically lead to the activities being resumed. One participant described how he had improved his ability to walk but that he still depended significantly on his wife fetching him things and had not taken up his previous habit of setting the table for dinner. He reflected that for him, the process of resuming daily activities required deliberate effort and feedback. Another perspective was that some activities were not in focus during the initial contact with the team but felt more important after a period of time. The lack of contact with the rehabilitation team could then be a barrier to finding strategies. An example of this was put forward in the second interview by one participant who felt that his rehabilitation had been too focused on physical function and that he felt left alone with resuming daily activities such as showering or cooking: *“Yes a little more of everyday eh tips and tricks so to speak. Cutting up a loaf of bread for example and things like that. We could have practised things like that a bit more”, (male, 45–66)*.

To contrast this view, some participants felt that they had come as far as they needed and that daily activities were running smoothly, although in a somewhat new manner and sometimes at a slower pace than before. As one person said:*"…I can do things that all normal people do (.) I can still do them but I do them a bit in my own way”, (male, 67–80)*.

## Discussion

The aim of this study was to explore and describe experiences of rehabilitation with F@ce 2.0 from the perspective of stroke survivors who have participated in the intervention. Participants talked about the significance of building a relationship with a competent rehabilitation team but also highlighted how SMSs can be supportive for goal achievement in stroke rehabilitation. Furthermore, the importance of having the team present in the home was discussed. These results will be discussed in relation to person-centredness, a central concept to the F@ce 2.0-intervention.

The importance of building a relationship between healthcare professionals and stroke survivors based on listening and understanding was a central finding in this study. Another important pillar of relationship building was the role of the healthcare professionals as experts who, in contrast to stroke survivors, come into the situation with knowledge and experience of rehabilitation. Both the significance of being listened to^3553^,^55^ and the role of healthcare professionals as knowledgeable with a self-evident role in guiding rehabilitation^35 54 56^ are recurring themes within the literature. Finding a balance in the interplay between the expertise of persons with stroke regarding life roles and preferences and the expertise of the team regarding stroke rehabilitation can be viewed as one of the fundamental challenges to person-centred goal setting in stroke rehabilitation. This balance may need to be adjusted over time, taking into consideration that increasing experience of life after stroke can render the person better equipped for active participation in goal setting later in the rehabilitation process.^57^ Using the patient’s lived experience is one of the two overall person-centred therapeutic strategies within the F@ce 2.0-intervention. To facilitate goal setting based on the experience of the person with stroke, the structured interview in the COPM is used. The COPM has been suggested as a tool to help healthcare professionals identify priorities of stroke survivors and link rehabilitation goals to daily activities.^34 58^ In the present study, participants talked about the goals set during the F@ce 2.0-intervention but sometimes described them more as instructions. This illustrates that even when striving towards person-centred goal setting, healthcare professionals and stroke survivors sometimes have conflicting perspectives. One explanation could be the aspiration of healthcare professionals to break overarching goals down into smaller achievable goals^54^ that then become more task-like. In the dialogue about rehabilitation goals, it may be helpful for stroke survivors if the connection between short-term rehabilitation goals and their overall goals for life after stroke is made clear.

In the present study, the ICT component was designed to support the achievement of personal goals through reminders and self-ratings. From the interviews, it was clear that most participants were aware of their rehabilitation goals. The interviews further indicated that SMSs can work both as reminders and motivators. This is in line with previous research where SMS reminders of goals are described by stroke survivors as helpful for goal achievement^41 42 59^ and self-management.^36^ The F@ce 2.0-intervention has a focus on enabling new experiences through the performance of valued daily activities. Messages are designed as reminders of the personal rehabilitation goals and are thus fully individualised. This way of basing ICT support on the already existing routine of setting goals as well as on readily available technology could be part of the solution for overcoming the challenge of making digital health accessible without requiring extensive resources. Previous research has disclosed that healthcare professionals view time constraints as a barrier for digital health^16 60^ and there may be a risk that the development of digital health will be driven towards standardised solutions. In the context of ICT for stroke rehabilitation, there are examples of interventions using a predesigned set of messages while still aiming for individualisation by ensuring that messages align with the person’s own goals.^59^ Others have argued that personalisation of ICT is too costly and demanding given the current healthcare system.^41^ This may, however, lead to a movement away from person-centred rehabilitation.

Results from the present study further indicate that daily ratings performed by the person with stroke may hold a potential for making rehabilitation more tangible. The importance of being aware of one’s progress has been highlighted in previous research focusing on the experience of stroke survivors.^55^ There were, however, conflicting reports regarding the realisation of this potential in the present study. While some participants talked about the positive aspects of reflecting on their goals in conjunction with answering the afternoon SMS, others did not see the SMSs as contributing to rehabilitation. The daily ratings sent from participants to the healthcare professionals were designed to act as an indicator of the need to adjust strategies or renegotiate goals to ensure a person-centred process. Even though some participants mentioned that their ratings had been persistently high or low, none of the participants reported that goals were adjusted during the intervention period. Participants’ statements accentuate that what is perceived as meaningful and relevant may change with time and experience of life after stroke. In line with this, it has been pointed out that the process of goal formulation should be seen as cyclic rather than linear.^61^ Such an ongoing person-centred process requires communication between the person with stroke and the rehabilitation team, where goals and strategies are revisited and evaluated to ensure a focus on what is relevant at present.

Another aspect discussed by participants in relation to ICT was the SMSs as a form of contact with the team and sometimes even as a sort of presence in between team visits. This is in line with previous research reporting that stroke survivors see digital solutions as one possibility for meeting their need of support in ongoing rehabilitation.^62^ There was, however, an ambiguity in reflections around ICT support, and all participants emphasised the significance of personal meetings with the team. The team was depicted as invoking a sense of safety and serving as a source of motivation in performing activities. Moreover, the meetings with the team were discussed as important in finding strategies for personally relevant daily activities. The results from the present study thus indicate that, although ICT can be helpful, it should be seen as a complement in stroke rehabilitation. Previous research has found that healthcare professionals view the use of ICT as a possible means to enable transparent communication about rehabilitation goals as well as a way of increasing access to information about stroke.^63 64^ Other research has, however, highlighted the role of healthcare professionals in digital health as motivators increasing adherence to self-led training,^35^ indicating the need to integrate digital resources and personal meetings, especially for people with moderate to severe stroke.^65^ In a changing and increasingly digitalised world, the development of digital health needs to focus on maintaining or increasing accessibility without losing the aspect of person-centredness in rehabilitation.

### Methodological considerations

This study was based on individual interviews with 12 stroke survivors who had participated in the F@ce 2.0-intervention. Interviews were carried out at two different time points, giving participants the opportunity to discuss not only their immediate experience of the intervention but also reflect back several months later. Participants differed in terms of gender, age and living conditions and contributed with a variety of experiences that were both overlapping and contrasting, painting a complex picture of partaking in ICT-supported person-centred rehabilitation. Due to the dependency on the overall research programme, including additional participants for the purpose of further refining themes was not possible. This is a limitation since inclusion of additional participants could have added additional perspectives. Specifically, only two participants were still of working age. During analysis, the researchers were involved in an ongoing discussion, refining codes and problematising the suggested themes while also going back and forth between codes and the original interviews. The heterogeneous professional backgrounds of the researchers as well as the differences in experience of qualitative research led to in-depth discussions on concepts reflected by themes, such as strategies and goals. Engaging in this process was vital to ensure reflexivity in the interpretation process and a comprehensive exploration of each proposed theme in relation to the study aim.^66^ The researchers acknowledge that their background within stroke rehabilitation has influenced their perspectives and invite the reader to critically appraise the results in relation to the research context as well as the specific intervention and the healthcare system within which it was offered.

## Conclusion

This study illustrates that ICT in the form of daily SMS reminders and ratings of rehabilitation goals was perceived as contributing to motivation and goal awareness in person-centred stroke rehabilitation. However, the results also point to the importance of personal contact in resuming daily activities after stroke, both for setting personally relevant goals and for finding and implementing strategies for goal achievement.

## Supplementary material

10.1136/bmjopen-2024-089147 online supplemental file 1

10.1136/bmjopen-2024-089147 online supplemental file 2

## Data Availability

Data are available upon reasonable request.
