# Supplementary material for: Experiences of F@ce 2.0: a person-centred intervention for home-based rehabilitation after stroke supported by digital technology — a qualitative study
Source: BMJ Open. 2025 Jul 16;15(7):e089147. doi: 10.1136/bmjopen-2024-089147 (PMC12273095; doi:10.1136/bmjopen-2024-089147)
Supplement: online supplemental file 1 [file bmjopen-15-7-s001.docx]

**Interview guide for people who have had a stroke and participated in F@CE**

The interview will begin with an open question such as;

**Can you tell us what a day has been like for you these weeks when you have participated in rehabilitation** (and had contact with the home rehabilitation team)? What could a day look like from the time you got up in the morning until you went to bed at night?

**Follow-up questions like:** How do you mean? Can you tell us more?

**About rehabilitation and rehabilitation goals**

- I would like to know more about your training and rehabilitation. Imagine that I don't know anything at all about rehabilitation/training after a stroke. Can you tell me what happened the first time you met? How has it been since? (Possibly ask about filming here).
- What are your biggest challenges during a day? How do you go about them?
- Is there anything that works better now compared to in the beginning?
- How do you think the rehabilitation has worked for you? What has been good and not so good? Would you like to change something?
- If there have been challenges, what have they looked like? Is there anything that has been difficult? In what way?
- How has the rehabilitation fitted into everyday life? Can you give examples of how daily life has been affected?
- Can you tell us what it was like in the beginning, just after the stroke? What about now? What has changed? Were there any specific turning points?
- Can you tell us about the goals of your rehabilitation
- Tell us about how you set goals
- Tell us about how you came up with this particular goal (are you satisfied with the goals - is X more important for you to do - would something else have been more important?)
- Tell us about how you and the team worked to achieve the goal - what strategies have you evaluated and changed

**About receiving support via text message**

- Tell us about the text messages. What has it been like to receive text messages?
- Can you show me some of the text messages on your phone?
- What significance have the reminders had for you? In what way has it been an asset or vice versa?
- Tell me how "performing" training towards your goal went in the beginning and how it went after a while.
- How has it worked with time to carry out these activities/training?
- How has it been to have to rate how today's training worked?
- Tell us about your thoughts when you performed the daily ratings. What did it mean when you felt that things had worked well? When things had gone badly, what did that mean?
- How do you think the technology has worked? This thing with receiving and responding to text messages.
- Have you received enough support with the text message?
- Has anyone shown you how you can go in and look at your previous answers?
- Have you had anyone close to you who has been involved? What has that involvment looked like? Can you tell us more? If not cohabiting/married – has anyone else still been involved?

**About the contact with the rehab team**

- Can you tell me what contact you have had with the rehabilitation team (OT, PT, SLT etc). What has it looked like? Who from the team have you met? How did you perceive the support you received? Is there any particular experience about the support you received that you would like to tell us about?
- How have you been able to convey your wishes to the rehabilitation team?
- Are there situations, contexts that you spontaneously think of regarding rehabilitation? What happened?

**About strategies**

- What do you think about the future if you encounter new challenges in everyday life? (Have you learned anything that you can use?) Are you doing anything different now compared to when you started rehabilitation? Can you give an example of a situation when a problem has arisen and you have solved it?
- If someone you know were to have a stroke. Would you recommend this type of rehabilitation?

SUMMARY

- Want to add something?

- May I contact you again

- How has the intervention worked, on a scale of 1-10 where 1 is that it has worked very badly and 10 is that it has worked in the best possible way.
