# Supplementary material for: Experiences of F@ce 2.0: a person-centred intervention for home-based rehabilitation after stroke supported by digital technology — a qualitative study
Source: BMJ Open. 2025 Jul 16;15(7):e089147. doi: 10.1136/bmjopen-2024-089147 (PMC12273095; doi:10.1136/bmjopen-2024-089147)
Supplement: online supplemental file 2 [file bmjopen-15-7-s002.docx]

**Interview guide for people who have had a stroke and participated in F@CE**

**Listen to interview 1 – follow up on specific questions for each person:**

**Follow-up questions like:** How do you mean? Can you tell us more?

**Life after stroke**

- If you think back on your everyday life – what was it like in the beginning after your stroke, what was it like when we met four months ago and how do you handle your everyday life now? Do you think there is anything that works better in the beginning and what do you still think is a challenge? When we met six months ago, you said...

……………………………………………………………………………………………………………………………………………………………………

- What do you consider to be the biggest change (for the better or for the worse?)
- How do you handle everyday life together with your family? How has stroke affected your life together? Can you tell us about how and what it has affected?
- How do you manage the activities that you need to do at home? Has it changed in any way?
- Is there anything that you have stopped doing because of your stroke?
- Is there anything that you stopped doing but have now started doing again? Withdrawn? How did you go about resuming it?
- Do you have any new habits/are you doing something new because of your stroke? Tell.
- Is there anything that works better now compared to in the beginning?
- Has anything special happened that has meant a change during your rehabilitation?
- When was the last time you did something that was a challenging situation/activity for you? What did you do then? How did you solve it?
- Is there anything else in your everyday life that you find challenging?
- If you are faced with something that is difficult to achieve but that you want to do – what do you think?
- What do you think about the future if you encounter new challenges in everyday life? (Is there anything that you have learned to use during this period since you had your stroke to solve a problem that arises in everyday life? Have you learned anything that you can use?)
- Are there any people who have been particularly important to you during this period? Can you tell me how, maybe describe some situation (if the Team is mentioned)

**Rehabilitation**

- When we last met, we talked about your rehabilitation/training after your stroke. Can you tell us how it has continued since we met?

--------------------------------------------------------------------------------------------------------------------------------------------------------------------------------------------------------------------------------------------------------------------------------------------------

- Can you tell me what contact you have had with the team now that came to your home (the neuro team/stroke team/home rehab team).
- Are you still in touch with them?
- How did you perceive the team's efforts? Is there any particular experience that you would like to tell us about?
- Have you received any other interventions/any other type of rehabilitation? Tell me about them!
- Have there been challenges, something that you have experienced as difficult with the rehabilitation, what have they looked like?
- How have the interventions you received/rehabilitation fit into your everyday life? Can you give examples of how everyday life has been affected?
- Part of your rehabilitation has been to plan what you want to improve. Can you tell us about what/what activities or situations you have aimed to manage?
- Do you have something now that you aim for? How do you get there? Give examples.
- When we last met, you had just stopped receiving text messages with reminders of your goals. When you look back on that period now – what was it like to receive text messages? What significance did it have? In what way (if anything) did it affect your rehabilitation and your everyday life?
- How do you think the rehabilitation as a whole has worked for you? What has been good and not so good? Is there anything that you would have liked to do differently?
- If someone you know were to have a stroke. Would you recommend this type of rehabilitation?

SUMMARY

You have now told me the following ...... Do you think I have understood this correctly? Is there anything you would like to clarify or add?

- Want to add something?

- May I contact you again?

- How has the rehabilitation worked, on a scale of 1-10 where 1 is that it has worked really badly and 10 is that it has worked in the best possible way.
