# Supplementary material for: Experiences of F@ce 2.0: a person-centred intervention for home-based rehabilitation after stroke supported by digital technology — a qualitative study
Source: BMJ Open. 2025 Jul 16;15(7):e089147. doi: 10.1136/bmjopen-2024-089147 (PMC12273095; doi:10.1136/bmjopen-2024-089147)
Supplement: Supplementary file 3 [file bmjopen-2024-089147.reviewer_comments.pdf]

PEER REVIEW HISTORY

BMJ Open publishes all reviews undertaken for accepted manuscripts. Reviewers are asked to complete a checklist review form and are provided with free text boxes to elaborate on their assessment. These free text comments are reproduced below.

ARTICLE DETAILS

Title (Provisional)

Experiences of Face 2.0 – a person-centred intervention for home-based rehabilitation after stroke supported by digital technology: A qualitative study

Authors

Söderhielm, Kajsa; Tistad, Malin; Ytterberg, Charlotte; Guidetti, Susanne

---

VERSION 1 - REVIEW

**Reviewer**	**1**
**Name**	**Tse, Tamara**
**Affiliation**	**La Trobe University**
**Date**	**30-Aug-2024**
**COI**	**No competing interests**

Thank you for the opportunity to review your manuscript on the use of ICT as personalised medicine. Your work is valuable in helping researchers and clinicians assess the usefulness of text messaging as a complementary approach to stroke rehabilitation.

I have provided suggestions to enhance the clarity and quality of the manuscript, particularly in the methods and findings sections, see the attached PDF for details. Additionally, I have highlighted a few referencing issues.

Overall, the text is well-written, but some sections toward the end could benefit from further revision as highlighted in the PDF.

**** **The reviewer provided a marked copy with additional comments. Please contact the publisher for full details.** ****

**Reviewer**	**2**
**Name**	**Forsyth, Rowena**
**Affiliation**	**Faculty of Medicine and Health**

Date **11-Jun-2025**

COI **No competing interests**

This is a well written article on an interesting topic. The authors make clear claims for the usefulness of digital interventions to support stroke patients and use in combination with personalised care from professionals. It is well situated within the existing literature. Methods are thoroughly described. Results and conclusion are clear.

Some minor points to address:

- Manuscript page 4 line 11 – reference format to be updated from author name to numbered.

- Manuscript page 6 line 16 – 'gate' should be 'gait'

- Some claims in the results require additional quotes to support them:

o Page 8 line 18 "participants for whom communication with the team felt natural and flexible depicted how this contributed to a sense of satisfaction."

o Page 8 line 34-5 "Participants depicted how the team sought to pinpoint what activities felt most important to resume but also expressed feeling unsure about what was achievable and needed the teams' expertise".

o Page 120 line 8 "She depicted how this had led to discussions around different aspects of progress in rehabilitation".

VERSION 1 - AUTHOR RESPONSE

Reviewer Comments:

**Reviewer 1**	
I have provided suggestions to enhance the clarity and quality of the manuscript, particularly in the methods and findings sections, see the attached PDF for details. Additionally, I have highlighted a few referencing issues.<br><br>Overall, the text is well-written, but some sections toward the end could benefit from further revision as highlighted in the PDF.	Thanks for the valuable comments!<br><br>A detailed list of changes in response to comments can be found below. Please note that page and line numbers refer to the paging in the original document (in alignment with the system for comments used by reviewer 2).
Manuscript page 4 line 11 – referencing issue	The reference has been changed from author name to numbered.
Manuscript page 4, Methodology and methods section. This paragraph describing the methodology and methods requires clarification. At this stage it is not clear who the	The section has been updated with brief information regarding the role of the researchers and the participants. Further

participants are and the role of the researchers in the study. Perhaps an explanation that the exploratory qualitative method is nested within the larger clustered study would be helpful.	information is found in the sections “Study setting”, “Participants” and “Research team”.
Manuscript page 6, line 12. Which interview did they partake in?	Information has been added to clarify details regarding participants who participated in one interview only. (Updated manuscript page 7).
Manuscript page 7, line 31. Duplicated on p.8 (manuscript page 5, subheading Participants).	The content has been revised to avoid duplication of information between the Participants and Ethics subheadings.
Manuscript page 9, lines 6-15. It would be helpful to include a statement at the beginning of this section 'the interplay needed to set relevant goals' describing challenges when there was a mismatch between the focus of rehabilitation team and the participants at the later interview.	A statement has been added to the beginning of the section (updated manuscript page 9). The new sentence reads: Most remembered partaking in a dialogue on matters that felt essential at that point in time, but in the follow-up interviews there were examples of the teams not fully having understood the priorities of the stroke survivor.
Manuscript page 9, lines 39-40. This point appears slightly different to that just described. A quote may help to demonstrate this additional point.	A quote has been added to illustrate the point. (Updated manuscript page 10).
Manuscript page 9, lines 42-43. Some clarification of this statement is needed to remind the reader the participants had to report their progress by texting through a number representing their level of achievement.	A clarification has been added, and the text now reads: One thing discussed was that reporting back by rating performance of goal activities on a scale from one to five was lacking in nuance. (Updated manuscript page 10).
Manuscript page 10, lines 5-8. It would be helpful to have a participant quote to demonstrate this point.	A participant quote has been added to illustrate the discussion between the stroke survivor and the team. (Updated manuscript page 10).
Manuscript page 10, line 16. Please revise whether this is the correct term. It seems as though it was not about comfort but more about whether they had sufficient capabilities.	It is indeed correct that it was less about being comfortable with technology and more about knowing how to actually use it. The wording has therefore been changed and the new sentence reads: Although owning a mobile phone was a criterion for participation in the project, not all participants knew how to send and read SMSs. (Updated manuscript page 10)
Manuscript page 10, paragraph “The need for support in using a mobile phone”. The last two paragraphs of this section are quite wordy compared to the previous paragraphs and would benefit from a review of the written text.	We agree hand have shortened these two paragraphs, keeping the main message but leaving out some of the details. (Updated manuscript page 11)
Manuscript page 11, line 41. It would be helpful to have a quote to explain this a little more.	A quote has been added for further explanation. (Updated manuscript page 12)

Manuscript page 11, lines 44-46. The quote and the explaining text do not quite align. Please review.	The quote has been replaced with another quote from the same participant discussing the same issue, which aligns better with the text. (Updated manuscript page 12)
Manuscript page 12, lines 18-19. This point needs further clarity. I am not sure what you mean by 'nothing automatic'	We have changed the wording to clarify. The updated sentence reads: He reflected that for him the process of resuming daily activities required deliberate effort and feedback. (Updated manuscript page 12)
Manuscript page 12, line 22. Please revise the term 'lifted', I'm not sure what you mean by this.	We have changed the wording from “lifted” to the more appropriate “put forward”. (Updated manuscript page 13)
**Reviewer 2**	
Manuscript page 4 line 11 – reference format to be updated from author name to numbered	The reference has been changed from author name to numbered.
Manuscript page 6 line 16 (in table 1) – ‘gate’ should be ‘gait’	The wording has been changed to gait. (Table 1)
Some claims in the results require additional quotes to support them:<br>o Page 8 line 18 “participants for whom communication with the team felt natural and flexible depicted how this contributed to a sense of satisfaction.”<br>o Page 8 line 34-5 “Participants depicted how the team sought to pinpoint what activities felt most important to resume but also expressed feeling unsure about what was achievable and needed the teams’ expertise”.<br>o Page 120 line 8 “She depicted how this had led to discussions around different aspects of progress in rehabilitation”.	- A quote has been added to illustrate this claim. (Updated manuscript page 8)<br>- This paragraph is a short introduction to the upcoming section. A quote illustrating the point is already present further down in the text: ”*Yes I have managed to get them [my wishes] through so to speak but I have such poor knowledge about the stroke… (.) ....I just have the wish that they should (.) that we should do things that are good for the stroke*" (male, 67-80). (Updated manuscript page 9)<br>- A quote has been added to illustrate this claim. Updated manuscript page 10
